# A systematic synthesis of expert opinion on effective policies to tackle bacterial resistance worldwide

**DOI:** 10.1002/vms3.1127

**Published:** 2023-03-23

**Authors:** Ali Mostafaei, Hamid Akbari, Cecilia Stålsby Lundborg, Neda Kabiri, Nafiseh Vahed, Sakineh Hajebrahimi, Leila Doshmangir

**Affiliations:** ^1^ Research Center for Evidence‐Based Medicine A JBI Centre of Excellence, Tabriz University of Medical Sciences Tabriz Iran; ^2^ Faculty of Veterinary Medicine, Department of Clinical Sciences University of Tabriz Tabriz Iran; ^3^ Department of Global Public Health Karolinska Institutet Stockholm Sweden; ^4^ Research Center of Psychiatry and Behavioral Sciences Tabriz University of Medical Sciences Tabriz Iran; ^5^ Faculty of Management and Medical Informatics, Iranian Center of Excellence for Heath management Tabriz University of Medical Sciences Tabriz Iran

**Keywords:** bacterial resistance, opinion, policy options, systematic review, text

## Abstract

Actions that are taken to preserve effective antibacterial agents and eliminate transmission of resistant organisms are crucial to prevent a catastrophic postantibiotic era. In this systematic review, we searched and appraised relevant texts and expert opinions to determine effective strategies to tackle bacterial resistance worldwide. We considered expert opinions, consensus, current discourses, comments, assumptions or assertions and discussion papers published in English. We searched following databases for expert opinion‐based literature: MEDLINE, CINAHL, ISI Web of Knowledge, SCOPUS, Cochrane Central Register of Controlled Trials and World Health Organization (WHO). We extracted the textual data from texts using a standardised data extraction tool. Textual pooling was not possible, so the conclusions were presented in a narrative form. Eighteen texts were included in this review. The findings show that, the most repeated policies and strategies include implementing and strengthening bacterial resistance surveillance, developing national guidelines, improving public awareness; enhancing home and everyday life hygiene; improving prescribing patterns, improving laboratories capacity, promoting innovation and research in new drugs and technology and strengthening coordination. This review systematically gathered strategies that were recommended by textual publications. To our knowledge, this was the first systematic review of text and opinion in the field of bacterial resistance. These results can be used by policymakers, hospital managers, and governments, alongside the results of quantitative and qualitative systematic reviews.

## Graphical abstract

In this systematic review, we searched and appraised relevant texts and expert opinions to determine effective strategies to tackle bacterial resistance worldwide. This is a novel type of systematic review, in which, the opinions of experts that has an important role in evidence‐based policymaking, are gathered and assessed. The findings show that the most repeated policies and strategies include implementing and strengthening bacterial resistance surveillance, developing national guidelines, improving public awareness, enhancing home and everyday life hygiene, improving prescribing patterns, improving laboratories capacity, promoting innovation and research in new drugs and technology, and strengthening coordination. Policymakers, hospital managers and governments, alongside the results of quantitative and qualitative systematic reviews, can use these results.

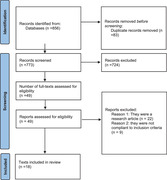

## 1 INTRODUCTION

The discovery of antibiotics has contributed to significant improvements in health and has led to decreased deaths from infectious diseases (Rubin & Samore, 2002). However, the high rate of antibiotic resistance due to the increased use of antibiotics, increased hospitalisation rate, and rising prevalence of health care‐associated infections, continues to be a worldwide threat. A part of antibiotics is used unnecessarily in agriculture, livestock and by physicians. Most of antibiotics, especially in low‐ and middle‐income countries, are utilised without prescription based on a correct diagnosis (Torres et al., 2021).

Without taking action to preserve effective bacterial agents and eliminate the transmission of resistant organisms, humans will soon risk reaching a postantibiotic era (Cohen, 1992). In addition, results of a systematic review indicated that antibiotic use without a prescription of correct diagnosis of a bacterial infection occurred worldwide and accounted for 19–100% of antimicrobial use outside of northern Europe and North America (Morgan et al., 2011). This study also revealed that antimicrobial‐resistant bacteria were common in settings with frequent nonprescription use (Morgan et al., 2011).

One of the most concerning factors related to microbial resistances is its high clinical and economic burden. The societal cost of antibiotic resistance attributable to each ambulatory antibiotic prescription in the United States was estimated to be 13$, of which 69% was due to the cost of hospitalisation (Michaelidis et al., 2016). Based on the results of a study, medical expenses of antimicrobial‐resistant infections ranged from 18,588$ to 29,069$ per patient; hospital stay days were longer for these patients being about 6.4−12.7 days; and societal costs were 10.7$−15.0$ (Roberts et al., 2009).

The World Health Organization (WHO) has recommended a global effort focusing on a multisectoral approach and collaboration among WHO, the Food and Agriculture Organization (FAO), and the World Organization for Animal Health (OIE) in the spirit of the ‘One Health’ approach (WHO, 2014). The reason for this collaborative approach is that people, animals and environmental and agricultural factors are the main drivers of antimicrobial resistance (Mcewen & Collignon, 2018). The One Health approach involves strong communication and coordination among the representatives of these three sections (Lammie & Hughes, 2016).

Due to the value of systematic review of expert opinion, the current systematic review of text and expert opinion was conducted to investigate expert's opinions, thoughts, and conclusions regarding bacterial resistance. This type of systematic review can stand alone or be used alongside quantitative or qualitative systematic reviews. To our knowledge, this was the first systematic review of text and opinion in the field of bacterial resistance.

## 2 METHODS

For our systematic review, we adhered to a unique methodology for the review of text and opinion (Mcarthur et al., 2015). In this type of systematic review, the opinions of experts, which has an important role in evidence‐based policymaking, are gathered and assessed. Evidence of text and opinion in this type of systematic review comes from expert opinions, consensus, current discourse, comments, assumptions or assertions that come in journals, magazines or reports (Mcarthur et al., 2015).

### 2.1 Inclusion criteria

#### 2.1.1 Population/type of participants (P)

The target audiences include patients using antibiotics, health care providers prescribing antibiotics, governments, policymakers and top health care managers.

#### 2.1.2 Intervention(s)/phenomena of interest (I)

This review considered publications that described reports, comments and viewpoints to combat bacterial resistance.

#### 2.1.3 Outcome (O)

The primary consequences in this review:

Policies that lead to lower rates of bacterial resistance.

Secondary consequences to this review:

Policies that have had the most significant impact regarding:

- Changes in behaviours of health care providers in prescribing unnecessary antibiotics.
- Change in cultural practices aiming to reduce the use of unnecessary antibiotics.

#### 2.1.4 Types of publications/narratives

We considered expert opinions, discussion papers, position papers and other forms of text, published in English. Technical reports, statistical reports and epidemiological reports were not included. Publications between 1 January 1990 and 30 January 2021 were eligible for inclusion.

### 2.2 Search strategy

A three‐step search strategy was utilised in each component of this review. An initial limited search of MEDLINE and CINAHL was undertaken, followed by an analysis of the text words contained in the title and abstract, and of the index terms used to describe the text. Having this information, we developed a search strategy that could be tailored for each information source. The reference list of all texts selected for critical appraisal was screened for additional publications. The full search strategy for databases is listed below.

The databases that were searched for expert opinion‐based literature, included MEDLINE, CINAHL, ISI Web of Knowledge, SCOPUS, Cochrane Central Register of Controlled Trials and WHO. The search for unpublished literature included ProQuest dissertations and thesis and the Networked digital library of thesis and dissertations (NDLTD). The following journals were also searched: *Journal of Health Politics*, *Health Affairs*, *Health Policy*, *Journal of Health Services Research and Policy*, *Health Policy and Planning*, *Journal of Public Health Policy*, *Bulletin of World Health Organization* and *Global Public Health*. A list of search terms and connectors used in this review can be found in Appendix 1.

### 2.3 Assessment of methodological quality

Textual papers selected for retrieval were assessed by two independent reviewers (NK and HA) for methodological quality using standardised critical appraisal instruments. This tool has six questions: Is the source of opinion clearly identified? Does the source of opinion have standing in the field of expertise? Are the interests of the relevant population the central focus of the opinion? Is the stated position the results of an analytical process and is this logic in the opinion expressed? Are there references to the extant literature? Is any incongruence with the literature/sources logically defended? Any disagreements between the reviewers were resolved through discussion, and in some cases, they were referred to the third reviewer. The reviewers considered the papers with a score of four and above as high‐quality paper.

### 2.4 Text selection

Following the search, all identified citations were collated and uploaded into Endnote software, and duplicates were removed. Titles and abstracts were screened by two independent reviewers (NK and HA), considering the inclusion criteria. Texts that met the inclusion criteria were retrieved in full. Full texts that did not meet the requirements of the inclusion criteria were excluded. Included texts underwent a process of critical appraisal. Disagreements between reviewers were resolved through discussion or with a third reviewer (SH).

### 2.5 Textual data extraction

Textual data were extracted from included texts in the review using a standardised data extraction tool, which includes specific details about the policies, populations, context and type of text.

### 2.6 Textual data synthesis

The aim of textual synthesis is to establish synthesised findings by bringing together key conclusions drawn from included texts. The conclusions were presented in narrative form. Two of the reviewers (NK and LD) read and re‐read the conclusions in order to identify similarities that can then be used to create categories of more than one finding. We used content analysis (Stemler, 2015) to categorise the findings into themes, counting and converting these themes into frequencies to identify dominant policies to tackle microbial resistance across a number of included texts.

## 3 RESULTS

**FIGURE 1:**
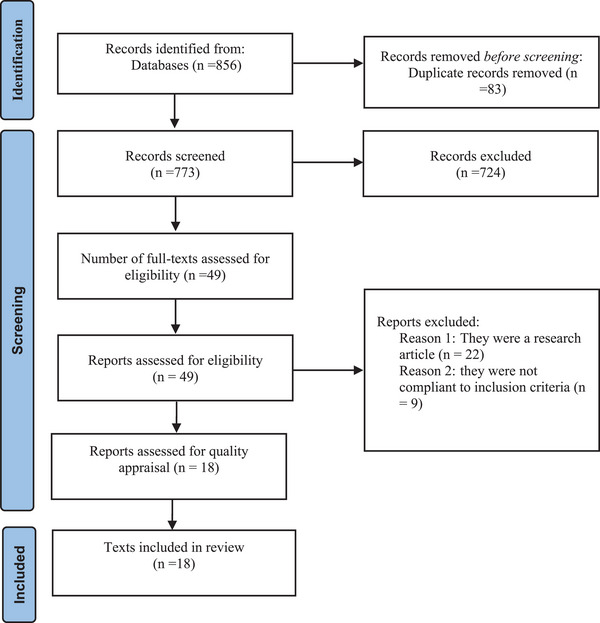
PRISMA flow diagram.

The primary search yielded 856 texts. After removing duplicates using bibliographic software (Endnote), 773 records remained. Title and abstract screening reduced this record to 49 documents. Of these, 31 were excluded due to noncompliance with the inclusion criteria. Finally, 18 texts were included in the review based on inclusion/exclusion criteria and methodological quality assessment. After full text review, the most common reasons for exclusion were as follows: (1) the document question did not meet the aim of systematic review and (2) they were a research article. Figure 1 is a PRISMA 2020 flow diagram of the study selection and inclusion process. Based on the predetermined method for quality assessment, all of the included documents had high quality and were included in the study.

### 3.1 Main characteristics of included texts

The included documents provided texts and expert opinions related to policies in order to determine effective strategies to tackle bacterial resistance worldwide. Except for one document, which was published in 1996 (Goldmann, 1996), others were published after 2000. Five of these documents were WHO reports (Thomas, 2014; WHO, 2015; WHO, 2001; WHO, 2011; WHO, 2016), two were policy papers (Dika et al., 2015; Walia et al., 2019), two were round table discussions (Balkhy et al., 2018; Balkhy et al., 2018) and two were commentaries (Tartari et al., 2017; Touraine, 2016). Others were the Centre of Disease Control (CDC) report (WHO, 2013), meeting report (Yam et al., 2019), consensus statement (Goldmann, 1996), viewpoint (Gross & Patel, 2007), poster (Turnidge et al., 2016), presidents page (Yewale, 2014) and position paper (Maillard et al., 2020). Represented population in the documents were governments, policymakers, hospital managers, health workers, prescribers, hospitals, patients and the general community. Of the 18 included texts, 15 mentioned the context of the published text. Of these, five were international documents (Thomas, 2014; WHO, 2015; WHO, 2001; WHO, 2011; WHO, 2016), two were Gulf cooperation council countries' documents (Balkhy et al., 2018; Balkhy et al., 2018) and two were published in the United States (WHO, 2013; Maillard et al., 2020) and India (Walia et al., 2019; Yewale, 2014). Others were published in Albania (Dika et al., 2015), France (Touraine, 2016) and Australia (Turnidge et al., 2016). The last one was published in the Asia Pacific region (Yam et al., 2019). The full characteristics of the included studies are indicated in Appendix 2.

### 3.2 Review findings

The process of content analysis yielded 132 themes. Five target audiences and settings were found based on the results of included text. Their audiences and settings were governments and policymakers, clinical settings and laboratories, prescribers, dispensers, health care providers, the public and veterinarians. Based on the results of content analysis, 13 core policies were identified, the most frequent of which across the 18 texts were as below: strengthening national surveillance of antibiotic resistance, improving prescribing patterns, improving public awareness, providing leadership and intersectoral collaboration, promoting and implementing national guidelines for antimicrobial treatments, fostering innovation and research on new drugs, enhancing infection prevention and control and regulating and promoting antimicrobial use in animal husbandry. Table 1 summarises the policies of all documents.

**TABLE 1:** Core policies for tackling bacterial resistance.

Target audience and setting	Policies
Governments and policymakers	Implementing and strengthening national surveillance of bacterial resistance Improving public awareness about the appropriate use of antimicrobial medicines and personal hygiene Strengthening national leadership, team working and collaboration among different sectors (human and animal) of the community Promoting innovation and research in new drugs and technology
Clinical settings and laboratories	Establishing infection prevention and control programs Establishing committees to control antibacterial use in hospitals Improving laboratory capacities to ensure quality assurance of diagnostic tests, microbial identification, susceptibility tests and timely reporting of results
Prescribers, dispensers and health care providers	Improving prescription patterns Developing and implementing national guidelines of clinical practice (diagnostic and treatment) Improving hand hygiene
Public	Enhancing home and everyday life hygiene
Veterinarians	Creating national systems to monitor antibacterial usage in food animals Developing guidelines for veterinarians about antibacterial use in food animals

## 4 DISCUSSION

This systematic review was conducted to search and appraise relevant texts and expert opinions, in order to suggest effective policies to tackle bacterial resistance.

Our findings showed that the most repeated policies and strategies in the documents were strengthening national surveillance of antibiotic resistance, improving prescribing patterns, improving public awareness, providing leadership and intersectoral collaboration, promoting and implementing national guidelines for antimicrobial treatments, fostering innovation and research on new drugs, enhancing infection prevention and control and regulating and promoting antimicrobial use in animal husbandry.

Surveillance systems as low‐cost tools to detect the emergence of antibacterial resistance are necessary for appropriate treatments and recommendations on antibacterial use (Blomberg et al., 2004; Cole et al., 2011). Results of a surveillance program in Norway showed that the prevalence of resistance was low in Norway in comparison with other countries; however, the use of antibiotics is low in this country. Norway has three systems for surveillance: The Norwegian Surveillance System for Communicable Diseases (MSIS), the Norwegian Surveillance System for Antimicrobial Drug Resistance (NORM) and the European Antimicrobial Resistance Surveillance System (EARSS) (Simonsen, 2009). In Thailand, a surveillance program has led to a cost‐effective approach to the treatment of diseases without risk of resistance (Shrivastava et al., 2016). Another antimicrobial surveillance system designed in the Netherlands has provided important data for policymakers, supporting the development of empirical antibiotic therapy guidelines and facilitating research (Altorf‐Van Der Kuil et al., 2017). In Sweden, the antibiotic resistance surveillance program relies on the results from the clinical, microbiological laboratories, the methods that are recommended by Nordic Committee on Antimicrobial Susceptibility Testing (NordicAST) (Aspevall et al., 2019).

Based on the Global Action Plan that was endorsed in May 2015 by the World Health Assembly, all countries were requested to develop their national action plans to tackle antimicrobial resistance (WHO, Global Action Plan on Antimicrobial Resistance, 2015). Results of the current study indicated that there should be well‐designed national and international policy decisions influenced by the political and social will to interrupt the increasing trend of bacterial resistance. Some countries, such as the United States (Spellberg et al., 2011), the United Kingdom (Kessel & Sharland, 2013), India (WHO, Global Action Plan on Antimicrobial Resistance. 2014) and China (Wang et al., 2016), have published such action plans. The development of such policies without engaging different stakeholders results in failure, as this issue is multifaceted and needs a broad range of stakeholder engagement and cooperation (Cui et al., 2017). In addition, it seems to be helpful to implement these guidelines with financial incentives.

One of the included documents in the current review was the ‘WHO global strategy for containment of antimicrobial resistance’, in which advocacy, education, management, and regulation of drug use were provided (WHO, 2001). One study assessed the advantages of this global report including, simple and comprehensive interventions provided in the program, and its generic framework, which can be applied to specific diseases such as HIV and malaria (Simonsen et al., 2004). The other report by WHO included in the current review, which was announced on 2011 world health day (WHO, 2011), had been used as an interview questionnaire in on study by The Western Pacific Regional Office of WHO (Lee & Wakabayashi, 2013). The results of the study showed that policymakers in the area of antimicrobial resistance were widely alert and knowledgeable about antimicrobial resistance.

The antimicrobial stewardship interventions, which were introduced in 2019 in India (Walia et al., 2019), were used to monitor the progress and implementation of the stewardship program in India between 2019 and 2021. The results indicated that the majority of tertiary hospitals implemented antimicrobial stewardship programs in their hospitals with the help of funding and capacity‐building activities from the Indian Council of Medical Research (Vijay et al., 2023). Another study used this policy document (Walia et al., 2019) to identify antibiotic prescription patterns after the decline of a major COVID‐19 wave at a dedicated COVID‐19 hospital in India. The results indicated that the strategies for antibiotic stewardship programs included developing guidelines for surgical prophylaxis, training physicians to categorise antibiotic prescriptions appropriately, and improving the electronic medical record system for improving prescription auditing (Sheikh et al., 2022). The secondary outcome of this paper was making changes in behaviours of health care providers in prescribing unnecessary antibiotics and our results implied ‘improving prescribing patterns’ as a core policy. Similarly, Saliba‐Gustafsson et al. (2017) designed a social marketing intervention in order to change general practitioners’ behaviour in antibiotic prescribing in Malta. Furthermore, medical students in the United States have an antimicrobial stewardship curricula in their preclinical and clinical years of school. They learn how to manage and diagnose common infections with correct treatment through some case‐based scenarios (Luther et al., 2013).

In the present study, we found that patient and public awareness is one of the policies for reducing bacterial resistance. Nepal and Bhatta (2018), in their recent systematic review, showed that self‐medication with antibiotics is very high in the WHO Southeast Asian region, which is one of the leading causes of microbial resistance. Jamhour et al. (2017) indicated that there was a significant correlation between self‐medication and educational level. Notably, the trend of self‐medication with antibiotics has changed since the novel coronavirus disease (COVID‐19) emerged. Preventive use of antibiotics against COVID‐19 was a result of a lack of understanding regarding the effects of antibiotics (Zhang et al., 2021).

As the results of the current systematic review show, there is a need for research on new medicines as a complement to antibiotics. Enioutina et al. (2017) indicated that natural products and herbal antimicrobial drugs could be advantageous in treatment. However, there is a need for more research on the efficacy and safety of these drugs (Enioutina et al., 2017). Dandekar proposed closer cooperation between pharmaceutical industries and basic research to turn research into new drugs (Dandekar & Dandekar, 2010).

### 4.1 Strengths and limitations of the study

This study provides an unprecedented opportunity to develop integrated efforts from experts’ opinions to better inform the design and improvement of bacterial resistance in the health system context, which expands the basis for evidence‐informed policymaking (Hasanpoor et al., 2018). This study has limitations. We adopted a systematic search strategy of texts and expert opinions to identify all related studies; however, some might have been missed.

## 5 CONCLUSIONS

Governments and policymakers can control bacterial resistance through some policies and strategies, the most frequent of which are setting up antibiotic surveillance programs, legislation for antibiotic prescription and improving public awareness. Also, governments have a leadership role and can strengthen the collaboration among a different sector of the community regarding the appropriate use of antimicrobials. There is a strong need for other qualitative and quantitative systematic reviews to be used alongside the results of current research by policymakers, hospital managers and government, who may wish to rely on evidence about effective policies and strategies to combat bacterial resistance.

## AUTHOR CONTRIBUTIONS

AM and SH supervised the whole study and revised the early draft of the manuscript. NV conducted the systematic search. HA and NK screened the included articles and assessed the methodological quality of studies. LD counselled the work and commented on the manuscript. NK extracted data and conducted the meta‐synthesis. HA prepared the early draft of the manuscript. CSL counselled the work, read many times and commented on the manuscript. All authors have read and agreed to the published version of the manuscript.

## CONFLICT OF INTEREST STATEMENT

The authors declare that the research was conducted in the absence of any commercial or financial relationships that could be construed as a potential conflict of interest.

### ETHICS STATEMENT

This study is a review study, so there is no need to ethical approval; however, ethics committee of Tabriz University of Medical Sciences approved the project (IR.TBZMED.VCR.REC.1398.251).

### PEER REVIEW

The peer review history for this article is available at https://publons.com/publon/10.1002/vms3.1127.

## Data Availability

data will be available in the appendices.

## APPENDIX 1 Full search strategy

**Table:**

**Search**	**Query**	**Results**
#1	Search: **“Drug Resistance, Microbial”[Mesh]**	168,294
#2	Search: **((“Microbial resistance”[Title/Abstract]) OR (((“Antibiotic Resistance”[Title/Abstract]) OR “Antimicrobial Drug Resistance”[Title/Abstract]) OR “Antimicrobial Drug Resistances”[Title/Abstract])) OR (“unnecessary antibiotic prescribing”[Title/Abstract])**	40,136
#3	Search: **(“Drug Resistance, Microbial”[Mesh]) OR (((“Microbial resistance”[Title/Abstract]) OR (((“Antibiotic Resistance”[Title/Abstract]) OR “Antimicrobial Drug Resistance”[Title/Abstract]) OR “Antimicrobial Drug Resistances”[Title/Abstract])) OR (“unnecessary antibiotic prescribing”[Title/Abstract]))**	187,990
#4	Search: **“Policy”[Mesh]**	160,880
#5	Search: **(policy[Title/Abstract]) OR polices[Title/Abstract]**	212,161
#6	Search: **(“Policy”[Mesh]) OR ((policy[Title/Abstract]) OR polices[Title/Abstract])**	320,393
#7	Search: **((“Drug Resistance, Microbial”[Mesh]) OR (((“Microbial resistance”[Title/Abstract]) OR (((“Antibiotic Resistance”[Title/Abstract]) OR “Antimicrobial Drug Resistance”[Title/Abstract]) OR “Antimicrobial Drug Resistances”[Title/Abstract])) OR (“unnecessary antibiotic prescribing”[Title/Abstract]))) AND ((“Policy”[Mesh]) OR ((policy[Title/Abstract]) OR polices[Title/Abstract]))**	1,876

## APPENDIX 2 Main characteristics of included texts

**Table:**

**Author (year)**	**Type of text**	**Target audiences of policy recommendations**	**Geographical setting**	**Content of the policy**
CDC 2013	CDC report‐USA	Governments	USA	Preventing infections and preventing the spread of resistance Tracking resistant bacteria Improving the use of today's antibiotics Promoting the development of new antibiotics and developing new diagnostic tests for resistant bacteria
Dika Q 2015	Policy paper	Policymakers and other national stakeholders	Albania	Strategic objectives: Strengthen national multisectorial coordination for the containment of antibiotic resistance Strengthen national surveillance of antibiotic resistance Promote national strategies for the rational use of antibiotics and strengthen national surveillance of antibiotic consumption Strengthen infection control and surveillance of antibiotic resistance in health care settings Prevent and control the development and spread of antibiotic resistance in the food chain Promote innovation and research on new drugs and technology Improve awareness, patient safety and partnership
Goldman D 1996	Consensus statement	Hospital leaders	–	The five strategic goals to optimise antimicrobial use: Optimising antimicrobial prophylaxis for operative procedures Optimising choice and duration of empiric therapy Improving antimicrobial prescribing by educational and administrative means Monitoring and providing feedback regarding antibiotic resistance Defining and implementing health care delivery system guidelines for important types of antimicrobial use The five strategic goals to detect, report, and prevent transmission of antimicrobial‐resistant organisms: Develop a system to recognise and report trends in antimicrobial resistance within the institution Develop a system to rapidly detect and report resistant microorganisms in individual patients and ensure a rapid response by caregivers Increase adherence to basic infection control policies and procedures Incorporate the detection, prevention, and control of antimicrobial resistance into institutional strategic goals and provide the required resources Develop a plan for identifying, transferring, discharging, and readmitting patients colonised with specific antimicrobial‐resistant pathogens
Gross PA 2007	Viewpoint	Patients with asymptomatic bacteriuria (ASB)	–	Developing performance measure for not treating asymptomatic bacteriuria in adults with antibiotic This measure should be adopted in pay‐for‐performance and pay‐for‐participation programs and should be part of the standards used in hospital accreditation
Tartari E 2017	Commentary	Health workers Hospital Chief Executive Officers and Administrators Policymakers IPC leaders	–	Health workers: ‘Clean your hands at the right times and stop the spread of antibiotic resistance’. Hospital Chief Executive Officers and Administrators: ‘Lead a year‐round infection prevention and control program to protect your patients from resistant infections’. Policymakers: ‘Stop antibiotic resistance spread by making infection prevention and hand hygiene a national policy priority’. IPC leaders: ‘Implement WHO's Core Components for infection prevention, including hand hygiene, to combat antibiotic resistance’.
Thomas G 2014	WHO Communications Officer report	Patients, health workers, policy makers	Global	People can help tackle resistance by: 1. Using antibiotics only when prescribed by a doctor 2. Completing the full prescription, even if they feel better 3. Never sharing antibiotics with others or using leftover prescriptions Health workers and pharmacists can help tackle resistance by: 1. Enhancing infection prevention and control 2. Only prescribing and dispensing antibiotics when they are truly needed 3. Prescribing and dispensing the right antibiotic(s) to treat the illness Policymakers can help tackle resistance by: 1. Strengthening resistance tracking and laboratory capacity 2. Regulating and promoting appropriate use of medicines Policymakers and industry can help tackle resistance by: 1. Fostering innovation and research and development of new tools 2. Promoting cooperation and information sharing among all stakeholders
Touraine M 2016	Comment	Governments	France	1. A program (**first program**) was adopted based on a public national awareness campaign. 2. Surveillance was also strengthened through the development of national reference centres and surveillance networks for antimicrobial use and resistance coordinated by the National Institute of Public Health 3. **Second national** plan adopted in 2006‐2010. Media campaigns aimed at the public highlighted the risk of antimicrobial resistance associated with bad prescribing practices. 4. An educational programme was also implemented in schools. 5. **Third program had three steps**: incentives were provided for better use of antimicrobials in hospitals; list of “critical antibiotics for human use” was requested; a new law was passed that empowers the health regional agencies to organise, monitor, and coordinate actions to reduce antimicrobial resistance at the regional level 6. In parallel, the first national plan for preserving the effectiveness of antibiotics in animals was launched.
Turnidge J 2016	Poster	Governments	Australia	A nationally coordinated and comprehensive surveillance system for antimicrobial use and antimicrobial resistance Antimicrobial stewardship programs
WHO 2015	WHO report (Worldwide country situation analysis)	Policymakers	Global	National plans: Surveillance and laboratory capacity Access to quality‐assured antimicrobial medicines Use of antimicrobial medicines Public awareness Infection prevention and control programs
WHO 2001	WHO Report	Patients and the general community, Prescribers, hospitals,	Global	**1. Patients and the general community** **Education** 1.1 Educate patients and the general community on the appropriate use of antimicrobials. 1.2 Educate patients on the importance of measures to prevent infection, such as immunisation, vector control, use of bed nets etc. 1.3 Educate patients on simple measures that may reduce transmission of infection in the household and community, such as hand washing, food hygiene etc. 1.4 Encourage appropriate and informed health care seeking behaviour. 1.5 Educate patients on suitable alternatives to antimicrobials for relief of symptoms and discourage patient self‐initiation of treatment, except in specific circumstances. **2. Prescribers and dispensers** **Education** 2.1 Educate all groups of prescribers and dispensers (including drug sellers) on the importance of appropriate antimicrobial use and containment of antimicrobial resistance. 2.2 Educate all groups of prescribers on disease prevention (including immunisation) and infection control issues. 2.3 Promote targeted undergraduate and postgraduate educational programmes on the accurate diagnosis and management of common infections for all health care workers, veterinarians, prescribers and dispensers. 2.4 Encourage prescribers and dispensers to educate patients on antimicrobial use and the importance of adherence to prescribed treatments. 2.5 Educate all groups of prescribers and dispensers on factors that may strongly influence their prescribing habits, such as economic incentives, promotional activities and inducements by the pharmaceutical industry. **Management, guidelines and formularies** 2.6 Improve antimicrobial use by supervision and support of clinical practices, especially diagnostic and treatment strategies. 2.7 Audit prescribing and dispensing practices and utilise peer group or external standard comparisons to provide feedback and endorsement of appropriate antimicrobial prescribing. 2.8 Encourage development and use of guidelines and treatment algorithms to foster appropriate use of antimicrobials. 2.9 Empower formulary managers to limit antimicrobial use to the prescription of an appropriate range of selected antimicrobials. **Regulation** 2.10 Link professional registration requirements for prescribers and dispensers to requirements for training and continuing education. **3. Hospitals** **Management** 3.1 Establish infection control programmes, based on current best practice, with the responsibility for effective management of antimicrobial resistance in hospitals and ensure that all hospitals have access to such a programme. 3.2 Establish effective hospital therapeutics committees with the responsibility for overseeing antimicrobial use in hospitals. 3.3 Develop and regularly update guidelines for antimicrobial treatment and prophylaxis, and hospital antimicrobial formularies. 3.4 Monitor antimicrobial usage, including the quantity and patterns of use, and feedback results to prescribers. **Diagnostic laboratories** 3.5 Ensure access to microbiology laboratory services that match the level of the hospital, e.g. secondary, tertiary. 3.6 Ensure performance and quality assurance of appropriate diagnostic tests, microbial identification, antimicrobial susceptibility tests of key pathogens, and timely and relevant reporting of results. 3.7 Ensure that laboratory data are recorded, preferably on a database, and are used to produce clinically and epidemiologically useful surveillance reports of resistance patterns among common pathogens and infections in a timely manner with feedback to prescribers and to the infection control program **Interactions with the pharmaceutical industry** 3.8 Control and monitor pharmaceutical company promotional activities within the hospital environment and ensure that such activities have educational benefit. **4. Use of antimicrobials in food‐producing animals** 4.1 Require obligatory prescriptions for all antimicrobials used for disease control in food animals. 4.2 In the absence of a public health safety evaluation, terminate or rapidly phase out the use of antimicrobials for growth promotion if they are also used for treatment of humans. 4.3 Create national systems to monitor antimicrobial usage in food animals. 4.4 Introduce prelicensing safety evaluation of antimicrobials with consideration of potential resistance to human drugs. 4.5 Monitor resistance to identify emerging health problems and take timely corrective actions to protect human health. 4.6 Develop guidelines for veterinarians to reduce overuse and misuse of antimicrobials in food animals.
WHO 2011	WHO recommendations on world health day	Governments	Global	5. The role of government. 6. Provide stewardship and Coordination. 7. Cost plans and mobilise resources. 8. Build partnerships with civil society. 9. Strengthen surveillance and laboratory capacity. 10. Establish AMR surveillance and monitoring systems. 11. Build laboratory capacity for rapid and reliable diagnostic testing. 12. Engage in regional and global surveillance networks. 13. Ensure uninterrupted access to essential medicines of assured quality. 14. Reinforce the system for supply of essential medicines. 15. Assure the quality of drugs according to international standards. 16. Regulate and promote rational use of medicines, including in animal husbandry, and ensure proper patient care. 17. Promote and enforce standard treatment guidelines. 18. Enforce prescription‐only use of antimicrobials. 19. Promote education on antimicrobial medicines and their use. 20. Reduce antimicrobial use in food producing animals. 21. Work to reduce financial incentives that encourage irrational use of medicines. 22. Reduce use of antimicrobials in food producing animals. 23. Provide national leadership and promote intersectoral collaboration. 24. Create and enforce an enabling regulatory framework. 25. Strengthen surveillance and monitoring. 26. Promote education and training on antimicrobial use in food producing animals. 27. Reduce the need for antimicrobials through better animal husbandry. 28. Enhance infection prevention and control. 29. Ensure availability of IPC programmes across the spectrum of health care. 30. Foster basic IPC standards in congregate settings. 31. Promote standard IPC measures and provide education on IPC in the community setting. 32. Foster innovations and research and development for new tools. 33. Improve the use of Current diagnostics and antimicrobials. 34. Create incentives for new product development. 35. Enable rapid regulatory processes for new tools and equitable access.
WHO 2016	Meeting Report	Policymakers	–	Development, dissemination and implementation of global antimicrobial resistance surveillance
Yewale V 2014	Presidents page	Governments	India	Developing and disseminating National Antibiotic Guidelines for Children 2014 – The IAP‐ICMR document Educating doctors—both paediatricians and others—and public on rational antibiotic practice Developing infection control guidelines for small hospitals and nursing homes, training the owners of such establishments and ensuring compliance by the members Collecting and collating data on antimicrobial resistance from the clinicians
Maillard JY 2020	Position paper	Patients and the general community	USA	Home and everyday life hygiene has the potential to reduce rates of infection and the need for antibiotic prescriptions.
Walia K 2019	Policy Document	Policymakers	India	Team organising Antibiograms and antibiotic policymaking Formularies and guidelines Training and certification of hospital staff Monitoring and reporting antibiotic use Goals and measurable outcomes Audit and feedback Interventions, education and awareness, and research
Yam ELY 2019	Meeting report	Governments	Asia Pacific Region	**Surveillance**: Harmonise sample and data collection procedures and enable access to samples and data for testing and mining. Adopt molecular epidemiology using whole‐genome sequencing to complement phenotypic studies for transmission dynamics. **Diagnostics**: Develop rapid diagnostic or point‐of‐care diagnostics for quick identification of pathogens and antimicrobial susceptibility. Develop nonpathogen‐based approaches that exploit human response signatures to complement existing pathogen‐targeted technologies. **Therapeutics**: Collaborate with regulatory agencies to shorten the timeline for drug development at all stages, from target discovery to clinical trials, thus reducing the cost of developing new AMR therapeutics. Develop new vaccine and antibiotic pipelines, and explore innovative approaches such as immunotherapy, phage therapy, drug repurposing, and combination therapy.
Balkhy H 2018	Round table discussion	Governments	Gulf Cooperation Council Countries	Collaboration among different sectors (human and animal) of the community and working as a team.
Balkhy H 2018	Round table discussion	Governments	Gulf Cooperation Council Countries	Surveillance, prevent transmission and prevent infections. Political commitment and leadership in decision‐making

## References

[vms31127-bib-0035] Altorf‐Van Der Kuil, W. , Schoffelen, A. F. , De Greeff, S. C. , Thijsen, S. F. , Alblas, H. J. , Notermans, D. W. , Vlek, A. L. , Van Der Sande, M. A. , & Leenstra, T. (2017). National laboratory‐based surveillance system for antimicrobial resistance: A successful tool to support the control of antimicrobial resistance in the Netherlands. Euro Surveillance, 22(46), 17–00062. 10.2807/1560-7917.ES.2017.22.46.17-00062 PMC5718398 29162208

[vms31127-bib-0036] Aspevall, O. , Wiener, V. , Nilsson, O. , & M, P. (2019). A report on Swedish Antibiotic Sales and Resistance in Human Medicine (Swedres) and Swedish Veterinary Antibiotic Re‐sistance Monitoring (Svarm). Sweden: Public Health Agency of Sweden and National Veterinary Institute.

[vms31127-bib-0021] Balkhy, H. H. , Zowawi, H. , Albatshan, H. A. , Alshamrani, M. M. , Aidara‐Kane, A. , Erlacher‐Vindel, E. , Alhawsawi, A. M. , Al‐Maani, A. S. , Al‐Abdely, H. M. , Balkhy, H. , Al‐Katheeri, H. A. , Al‐Fadhli, M. , Abdulrazzaq, N. M. , Al‐Khawaja, S. , Hawsawi, A. , Algwizani, A. , Amri, A. A. l. , Saedy, A. A. l. , Omran, A. , … Qunaibet, A. (2018). Antimicrobial resistance: A round table discussion on the “One Health” concept from the Gulf Cooperation Council Countries. Part One: A focus on Leadership. Journal of Infection and Public Health, 11(6), 771–777. 30396638 10.1016/j.jiph.2018.05.007

[vms31127-bib-0022] Balkhy, H. H. , Zowawi, H. M. , Alshamrani, M. M. , Allegranzi, B. , Srinivasan, A. , Al‐Abdely, H. M. , Somily, A. M. , Al‐Quwaizani, M. A. , Al‐Maani, A. S. , Al‐Abdely, H. M. , Balkhy, H. , Al‐Katheeri, H. A. , Al‐Fadhli, M. , Abdulrazzaq, N. M. , Al‐Khawaja, S. , Hawsawi, A. , Gwizani, A. A. l , Amri, A. A. l. , Saedy, A. A. l. , … Omran, A. (2018). Antimicrobial resistance: A round table discussion on the “One Health” concept from the Gulf Cooperation Council Countries. Part Two: A focus on Human Health. Journal of Infection and Public Health, 11(6), 778–783. 30396639 10.1016/j.jiph.2018.05.008

[vms31127-bib-0031] Blomberg, B. , Mwakagile, D. S. M. , Urassa, W. K. , Maselle, S. Y. , Mashurano, M. , Digranes, A. , Harthug, S. , & Langeland, N. (2004). Surveillance of antimicrobial resistance at a tertiary hospital in Tanzania. BMC Public Health [Electronic Resource], 4, 45. 15476559 10.1186/1471-2458-4-45 PMC526372

[vms31127-bib-0025] CDC (2013). Antibiotic resistance threats in the United States. USA: Center of Disease Control and Prevention 2013.

[vms31127-bib-0003] Cohen, M. L. (1992). Epidemiology of drug resistance: Implications for a post – Antimicrobial era. Science, 257(5073), 1050–1055. 1509255 10.1126/science.257.5073.1050

[vms31127-bib-0040] “Chennai Declaration” Team . (2014). “Chennai Declaration”: 5‐year plan to tackle the challenge of anti‐microbial resistance. Indian Journal of Medical Microbiology, 32(3), 221–228. 25008811 10.4103/0255-0857.129053

[vms31127-bib-0032] Cole, M. J. , Unemo, M. , Hoffmann, S. , Chisholm, S. A. , Ison, C. A. , & Van De Laar, M. J. (2011). The European gonococcal antimicrobial surveillance programme, 2009. Eurosurveillance, 16(42), 19995. 22027378

[vms31127-bib-0042] Cui, D. , Liu, X. , Hawkey, P. , Li, H. , Wang, Q. , Mao, Z. , & Sun, J. (2017). Use of and microbial resistance to antibiotics in China: A path to reducing antimicrobial resistance. Journal of International Medical Research, 45(6), 1768–1778. 29239248 10.1177/0300060516686230 PMC5805194

[vms31127-bib-0053] Dandekar, T. , & Dandekar, G. (2010). Pharmacogenomic strategies against microbial resistance: From bright to bleak to innovative. Pharmacogenomics, 11(9), 1193–1196. 20860457 10.2217/pgs.10.18

[vms31127-bib-0019] Dika, Q. , Duli, M. , & Kalo, I. (2015). Health policies on antibiotic resistance in Albania. European Scientific Journal, **11**(36).

[vms31127-bib-0052] Enioutina, E Yu. , Teng, L. , Fateeva, T V. , Brown, J. C. S. , Job, K. M. , Bortnikova, V. V. , Krepkova, L. V. , Gubarev, M. I. , & Sherwin, C. M. T. (2017). Phytotherapy as an alternative to conventional antimicrobials: combating microbial resistance. Expert Review of Clinical Pharmacology, 10(11), 1203–1214. 28836870 10.1080/17512433.2017.1371591

[vms31127-bib-0013] Goldmann, D. A. (1996). Strategies to prevent and control the emergence and spread of antimicrobial‐resistant microorganisms in hospitals. Jama, 275(3), 234. 8604178

[vms31127-bib-0027] Gross, P. A. , & Patel, B. (2007). Reducing antibiotic overuse: A call for a national performance measure for not treating asymptomatic bacteriuria. Clinical Infectious Diseases, 45(10), 1335–1337. 17968830 10.1086/522183

[vms31127-bib-0054] Hasanpoor, E. , Hajebrahimi, S. , Janati, A. , Abedini, Z. , & Haghgoshayie, E. (2018). Barriers, facilitators, process and sources of evidence for evidence‐based management among health care managers: A qualitative systematic review. Ethiopian Journal of Health Sciences, 28(5), 665–680. 30607082 10.4314/ejhs.v28i5.18 PMC6308777

[vms31127-bib-0050] Jamhour, A. , El‐Kheir, A. , Salameh, P. , Hanna, P. A. , & Mansour, H. (2017). Antibiotic knowledge and self‐medication practices in a developing country: A cross‐sectional study. American Journal of Infection Control, 45(4), 384–388. 28087169 10.1016/j.ajic.2016.11.026

[vms31127-bib-0039] Kessel, A. S. , & Sharland, M. (2013). The new UK antimicrobial resistance strategy and action plan. Bmj, 346(mar11 3), f1601. 23479662 10.1136/bmj.f1601

[vms31127-bib-0009] Lammie, S. L. , & Hughes, J. M. (2016). Antimicrobial resistance, food safety, and One Health: The need for convergence. Annual Review of Food Science and Technology, 7, 287–312. 10.1146/annurev-food-041715-033251 26772408

[vms31127-bib-0044] Lee, Y. , & Wakabayashi, M. (2013). Key informant interview on antimicrobial resistance (AMR) in some countries in the Western Pacific region. Globalization and Health, 9, 34. 23889997 10.1186/1744-8603-9-34 PMC3733822

[vms31127-bib-0048] Luther, V. P. , Ohl, C. A. , & Hicks, L. A. (2013). Antimicrobial stewardship education for medical students. Clinical Infectious Diseases, 57(9), 1366. 23893971 10.1093/cid/cit480

[vms31127-bib-0030] Maillard, J. Y. , Bloomfield, S. F. , Courvalin, P. , Essack, S. Y. , Gandra, S. , Gerba, C. P. , Rubino, J. R. , & Scott, E. A. (2020). Reducing antibiotic prescribing and addressing the global problem of antibiotic resistance by targeted hygiene in the home and everyday life settings: A position paper. American Journal of Infection Control, 48(9), 1090–1099. 32311380 10.1016/j.ajic.2020.04.011 PMC7165117

[vms31127-bib-0010] Mcarthur, A. , Klugárová, J. , Yan, H. U. , & Florescu, S. (2015). Innovations in the systematic review of text and opinion. International Journal of Evidence‐Based Healthcare, 13(3), 188–195. 26207851 10.1097/XEB.0000000000000060

[vms31127-bib-0008] Mcewen, S. A. , & Collignon, P. J. (2018). Antimicrobial resistance: A One Health Perspective. Microbiology Spectrum, **6**(2). 10.1128/microbiolspec.arba-0009-2017 PMC11633550 29600770

[vms31127-bib-0005] Michaelidis, C. I. , Fine, M. J. , Lin, C. J. , Linder, J A. , Nowalk, M. P. , Shields, R. K. , Zimmerman, R. K. , & Smith, K. J. (2016). The hidden societal cost of antibiotic resistance per antibiotic prescribed in the United States: an exploratory analysis. BMC Infectious Diseases, **16**(1). 10.1186/s12879-016-1990-4 PMC5101711 27825306

[vms31127-bib-0004] Morgan, D. J. , Okeke, I. N. , Laxminarayan, R. , Perencevich, E. N. , & Weisenberg, S. (2011). Non‐prescription antimicrobial use worldwide: A systematic review. The Lancet Infectious Diseases, 11(9), 692–701. 21659004 10.1016/S1473-3099(11)70054-8 PMC3543997

[vms31127-bib-0049] Nepal, G. , & Bhatta, S. (2018). Self‐medication with antibiotics in WHO Southeast Asian Region: A systematic review. Cureus, 10(4), e2428. 29876150 10.7759/cureus.2428 PMC5988199

[vms31127-bib-0012] Page, M. J. , Mckenzie, J. E. , Bossuyt, P. M. , Boutron, I. , Hoffmann, T. C. , Mulrow, C. D. , Shamseer, L. , Tetzlaff, J. M. , Akl, E. A. , Brennan, S. E. , Chou, R. , Glanville, J. , Grimshaw, J. M. , Hróbjartsson, A. , Lalu, M. M. , Li, T. , Loder, E. W. , Mayo‐Wilson, E. , Mcdonald, S. , … Mcguinness, L. A. (2021). The PRISMA 2020 statement: An updated guideline for reporting systematic reviews. Bmj, 372, n71. 33782057 10.1136/bmj.n71 PMC8005924

[vms31127-bib-0006] Roberts, R. R. , Hota, B. , Ahmad, I. , Scott Ii, R. D. , Foster, S. D. , Abbasi, F. , Schabowski, S. , Kampe, L. M. , Ciavarella, G. G. , Supino, M. , Naples, J. , Cordell, R. , Levy, S. B. , & Weinstein, R. A. (2009). Hospital and societal costs of antimicrobial‐resistant infections in a chicago teaching hospital: Implications for antibiotic stewardship. Clinical Infectious Diseases, 49(8), 1175–1184. 19739972 10.1086/605630

[vms31127-bib-0001] Rubin, M. A. , & Samore, M. H. (2002). Antimicrobial use and resistance. Current Infectious Disease Reports, 4(6), 491–497. 12433323 10.1007/s11908-002-0034-y

[vms31127-bib-0047] Saliba‐Gustafsson, E. A. , Borg, M. A. , Rosales‐Klintz, S. , Nyberg, A. , & StålsbyLundborg, C. (2017). Maltese Antibiotic Stewardship Programme in the Community (MASPIC): Protocol of a prospective quasiexperimental social marketing intervention. BMJ Open, 7(9), e017992. 10.1136/bmjopen-2017-017992 PMC5623537 28947463

[vms31127-bib-0046] Sheikh, S. , Vishwas, G. , Aggarwal, M. , Bhattacharya, S. , Kumari, P. , Parashar, L. , & Meshram, G. G. (2022). Antibiotic point prevalence survey at a tertiary healthcare hospital in India: Identifying strategies to improve the antibiotic stewardship program immediately after a COVID‐19 wave. Infection Prevention in Practice, 4(4), 100253. 36276168 10.1016/j.infpip.2022.100253 PMC9562613

[vms31127-bib-0034] Shrivastava, S. , Shrivastava, P. , & Ramasamy, J. (2016). Implementation of enhanced gonococcal antimicrobial surveillance program in Thailand. Indian Dermatology Online Journal, 7(6), 545–546. 27990400 10.4103/2229-5178.193920 PMC5134179

[vms31127-bib-0043] Simonsen, G. S. , Tapsall, J. W. , Allegranzi, B. , Talbot, E. A. , & Lazzari, S. (2004). The antimicrobial resistance containment and surveillance approach – A public health tool. Bulletin of the World Health Organization, 82(12), 928–934. 15654407 PMC2623104

[vms31127-bib-0033] Simonsen, G. (2009). Surveillance and prevalence of antimicrobial resistance in Norway. Tidsskrift for Den Norske Laegeforening, 129(7), 623–627. 19337330 10.4045/tidsskr.08.0012

[vms31127-bib-0038] Spellberg, B. , Blaser, M. , Guidos, R. J. , Boucher, H. W. , Bradley, J. S. , & Eisenstein, B. I. (2011). Combating antimicrobial resistance: policy recommendations to save lives. Clinical Infectious Diseases, 52(Suppl 5), S397–428. 21474585 10.1093/cid/cir153 PMC3738230

[vms31127-bib-0011] Stemler, S. E. (2015). Content analysis. In R. A. Scott & S. M., Kosslyn (Eds.), Emerging trends in the social and behavioral sciences (pp. 1–14). Wiley.

[vms31127-bib-0023] Tartari, E. , Abbas, M. , Pires, D. , & De Kraker, M. E. A. , Pittet, D. (2017). World Health Organization SAVE LIVES: Clean Your Hands global campaign—‘Fight antibiotic resistance—it's in your hands’. Clinical Microbiology and Infection, 23(9), 596–598. 28487167 10.1016/j.cmi.2017.04.021

[vms31127-bib-0014] Thomas, G. (2014). WHO's first global report on antibiotic resistance reveals serious, worldwide threat to public health. Geneva: WHO.

[vms31127-bib-0002] Torres, N. F. , Chibi, B. , Kuupiel, D. , Solomon, V. P. , Mashamba‐Thompson, T. P. , & Middleton, L. E. (2021). The use of non‐prescribed antibiotics; prevalence estimates in low‐and‐middle‐income countries. A systematic review and meta‐analysis. Archives of Public Health, 79(1), 2. 33390176 10.1186/s13690-020-00517-9 PMC7780654

[vms31127-bib-0024] Touraine, M. (2016). Tackling antimicrobial resistance in France. The Lancet, 387(10034), 2177–2179. 10.1016/S0140-6736(16)30356-7 27145702

[vms31127-bib-0028] Turnidge, J. , Meleady, K. , & Bell, J. (2016). Developing a national surveillance system for antimicrobial use and resistance in Australia: AURA. Infection, Disease & Health, 21(3), 123.

[vms31127-bib-0045] Vijay, S. , Ramasubramanian, V. , Bansal, N. , Ohri, V. C , & Walia, K. (2023). Hospital‐based antimicrobial stewardship, India. Bulletin of the World Health Organization, 101(1), 20–27A. 36593779 10.2471/BLT.22.288797 PMC9795386

[vms31127-bib-0020] Walia, K. , Ohri, V. C. , Madhumathi, J. , & Ramasubramanian, V. (2019). Policy document on antimicrobial stewardship practices in India. Indian Journal of Medical Research, 149(2), 180. 31219081 10.4103/ijmr.IJMR_147_18 PMC6563731

[vms31127-bib-0041] Wang, L. I. , Zhang, X. , Liang, X. , & Bloom, G. (2016). Addressing antimicrobial resistance in China: Policy implementation in a complex context. Global Health, 12(1), 30. 27267876 10.1186/s12992-016-0167-7 PMC4893878

[vms31127-bib-0037] WHO (2015). Global Action Plan on Antimicrobial Resistance.

[vms31127-bib-0016] WHO (2001). WHO global strategy for containment of antimicrobial resistance. Switzarland.

[vms31127-bib-0017] WHO (2011). Strategies to combat antimicrobial resistance: World Health Day.

[vms31127-bib-0007] WHO (2014). Antimicrobial resistance: Global report on surveillance: World Health Organization.

[vms31127-bib-0015] WHO (2015). Worldwide country situation analysis: response to antimicrobial resistance. Switzerland.

[vms31127-bib-0018] WHO (2016). Report of the 2nd Meeting of the Global AMR Surveillance System (GLASS) Collaborative Platform. Geneva, Switzerland: WHO .

[vms31127-bib-0026] Yam, E. L. Y. , Hsu, L. Y. , Yap, E. P. ‐H. , Yeo, T. W. , Lee, V. , Schlundt, J. , Lwin, M. O. , Limmathurotsakul, D. , Jit, M. , Dedon, P. , Turner, P. , & Wilder‐Smith, A. (2019). Antimicrobial Resistance in the Asia Pacific region: A meeting report. Antimicrobial Resistance & Infection Control, **8**(1). 10.1186/s13756-019-0654-8 PMC6921568 31890158

[vms31127-bib-0029] Yewale, V. N. (2014). IAP‐ICMR call to action to tackle the antimicrobial resistance. Indian Pediatrics, 51(6), 437–439. 24986274 10.1007/s13312-014-0429-5

[vms31127-bib-0051] Zhang, A. , Hobman, E. V. , De Barro, P. , Young, A. , Carter, D. J. , & Byrne, M. (2021). Self‐medication with antibiotics for protection against COVID‐19: The role of psychological distress, knowledge of, and experiences with antibiotics. Antibiotics (Basel), 10(3), 232. 33668953 10.3390/antibiotics10030232 PMC7996601

